# Supplementary material for: Weighted Gene Co-Expression Network Analysis and Support Vector Machine Learning in the Proteomic Profiling of Cerebrospinal Fluid from Extraventricular Drainage in Child Medulloblastoma
Source: Metabolites. 2022 Aug 5;12(8):724. doi: 10.3390/metabo12080724 (PMC9412589; doi:10.3390/metabo12080724)
Supplement: Supplementary file 1 [file metabolites-12-00724-s001.zip › metabolites-1827382-Supplement Figure.zip!/metabolites-1827382-Supplement Figure.pdf]

metabolites

MDPI

Article

Weighted Gene Co-Expression Network Analysis and Support Vector Machine Learning in the Proteomic Profiling of Cerebrospinal Fluid from Extraventricular Drainage in Child Medulloblastoma

Maurizio Bruschi [1],Xhuliana Kajana [1], Andrea Petretto [2], Martina Bartolucci [2], Marco Pavanello [3], Gian Marco Ghiggeri [4], Isabella Panfoli [5,†,*], and Giovanni Candiano [1,†]

Supplementary Figures

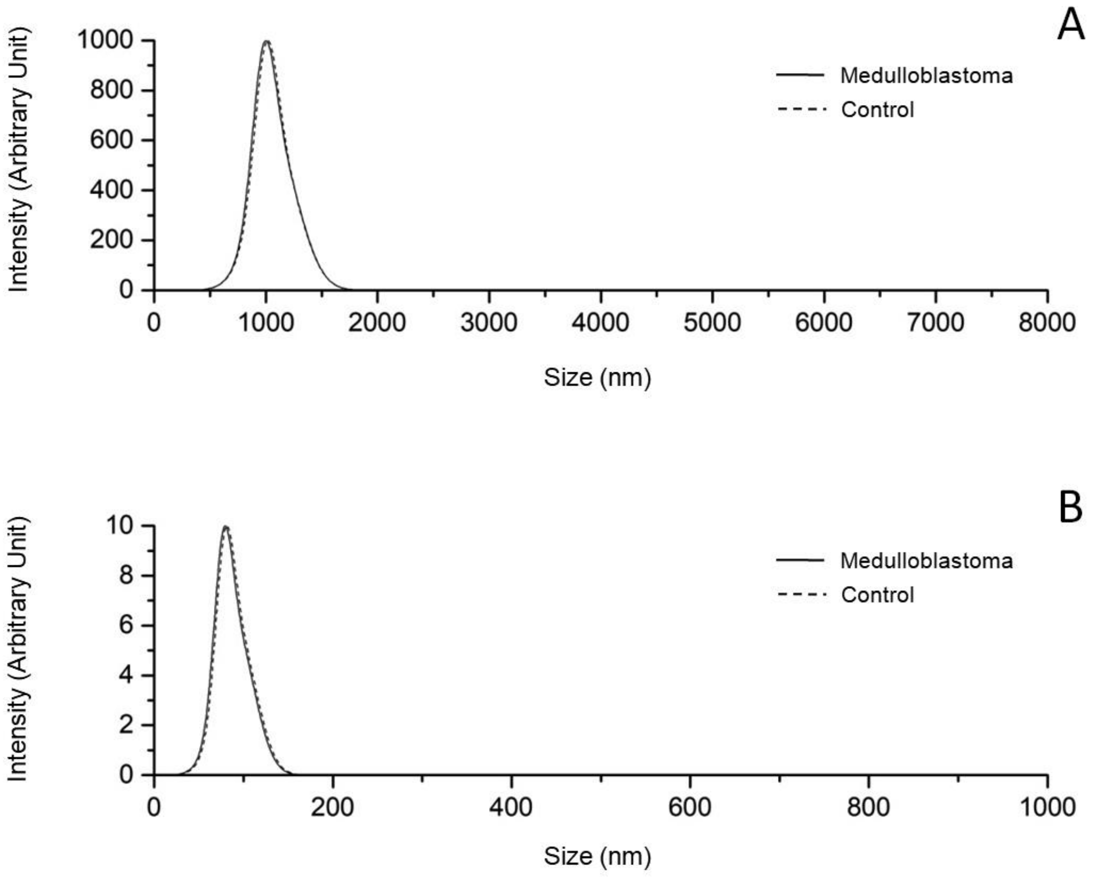

Figure S1. Characterization of extracellular vesicles purified in CSF from EVD of MB and control patients. Plots of **A**) microvesicle, and B) exosome size distribution, as evaluated by dynamic light scattering. Both plots show a Gaussian distribution profile with a mean peak at 1000 ± 75 nm, and 100 ± 5 nm for microvesicle, and exosome respectively. No statistical differences were observed between the microvesicle or exosome isolated in CSF from EVD of MB and control patients.

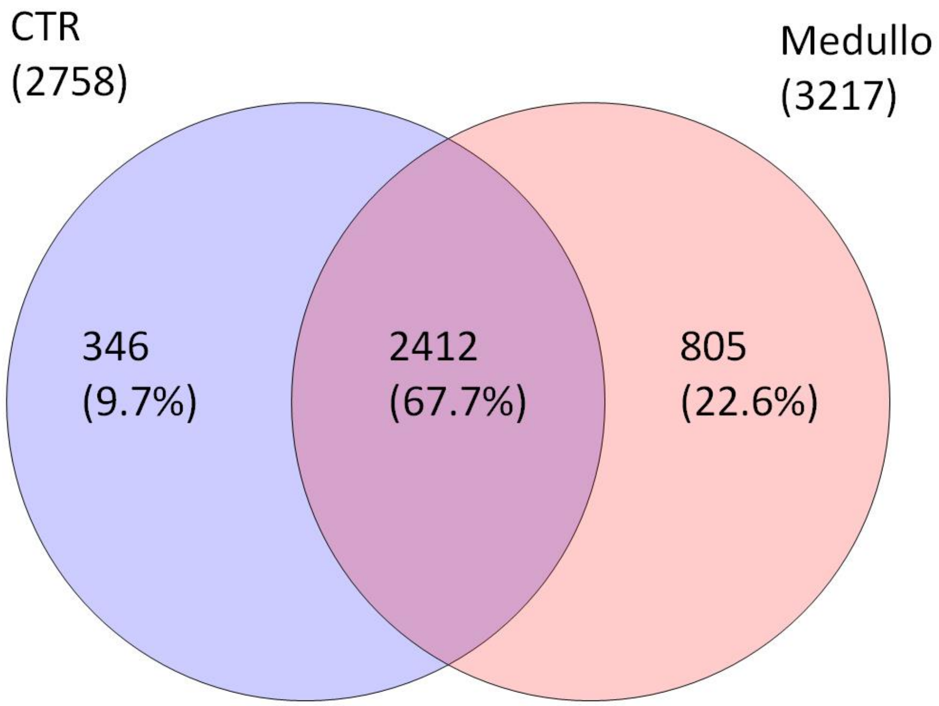

Figure S2. Venn diagram of total proteins identified in CSF from EVD of MB and control samples. Venn diagram shows common and exclusive proteins identified in all samples and fractions of Medulloblastoma (Medullo) and control (CTR) patients. The numbers represent the distinct proteins in the overlapping and non-overlapping areas**.**

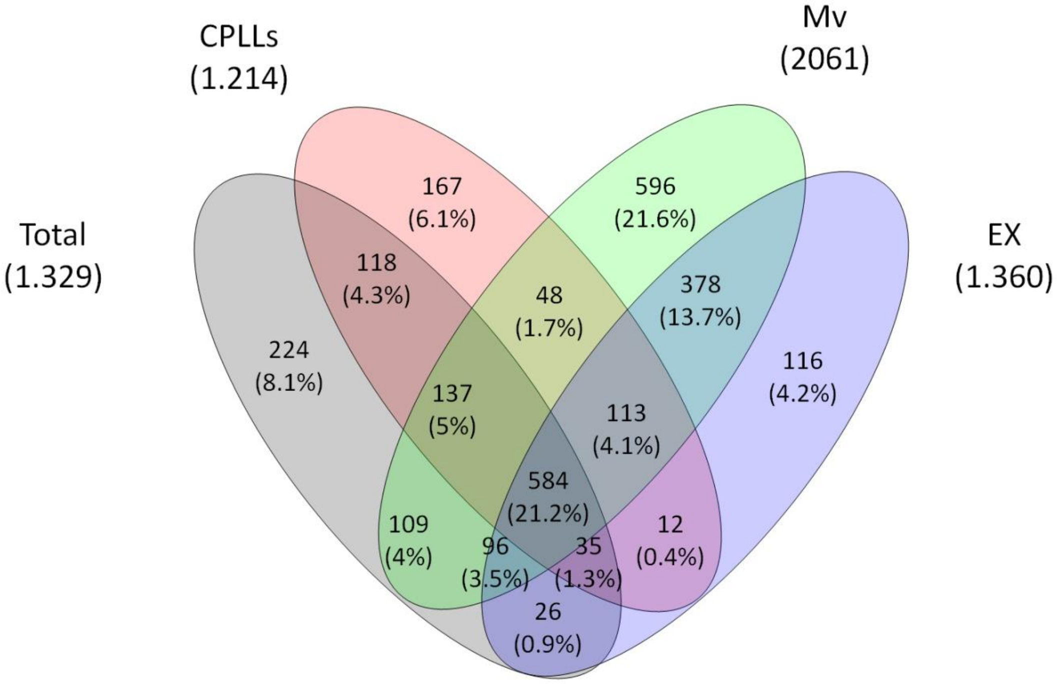

Figure S3. Venn diagram of total proteins identified in CSF from EVD of control samples. Venn diagram shows common and exclusive proteins identified in total CSF (Total), microvesicle (Mv), exosome (Ex) and proteins captured by peptide library beads (CPLLs) fractions of control patients. The numbers represent the distinct proteins in the overlapping and non-overlapping areas**.**

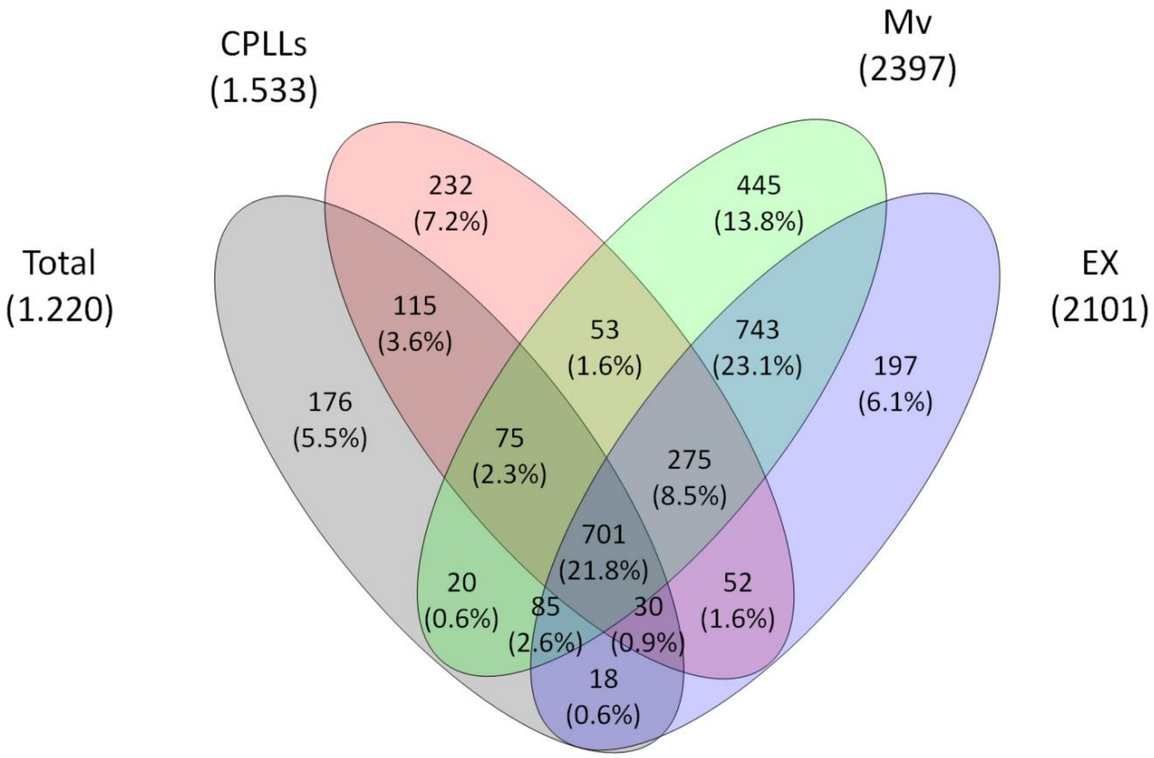

Figure S4. Venn diagram of total proteins identified in CSF from EVD of MB samples. Venn diagram shows common and exclusive proteins identified in total CSF (Total), microvesicle (Mv), exosome (Ex) and proteins captured by peptide library beads (CPLLs) fractions of patients with Medulloblastoma tumor. The numbers represent the distinct proteins in the overlapping and non-overlapping areas.

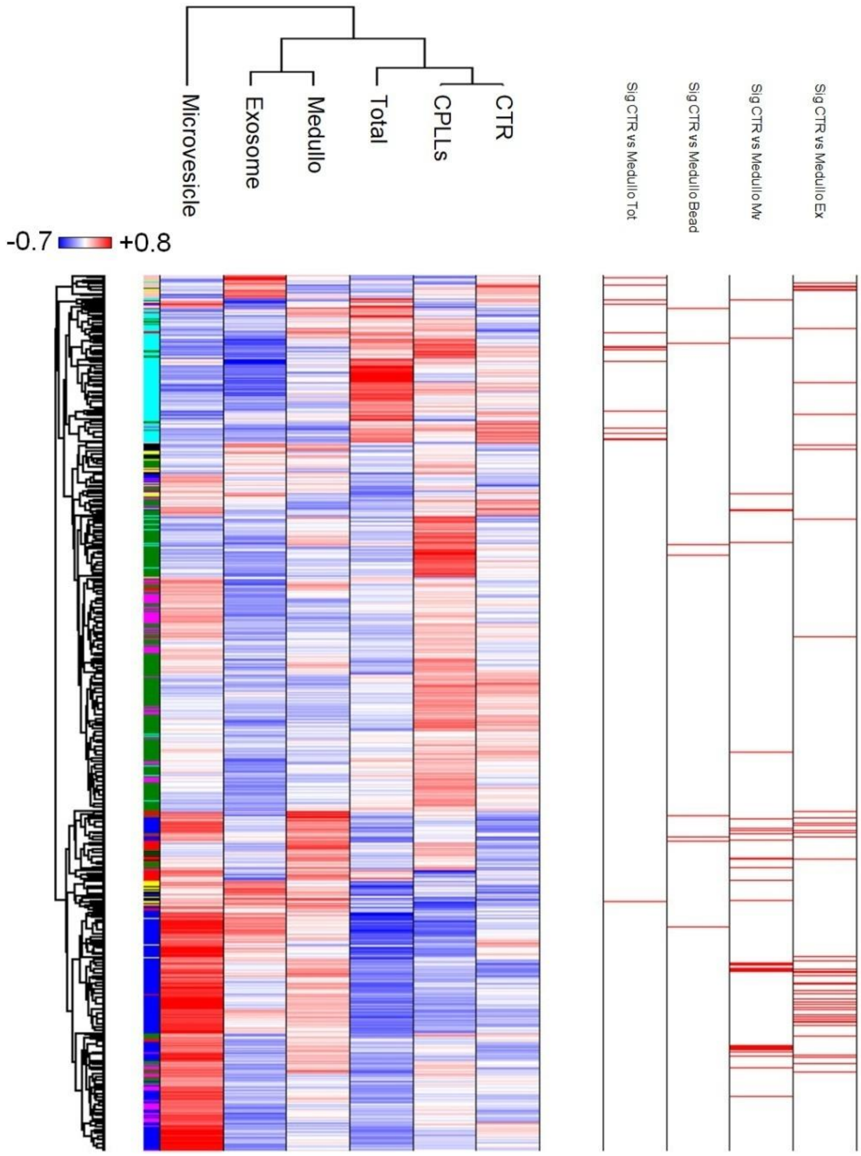

Figure S5. Weighted gene co-expression analysis of proteins identified in CSF from EVD of MB and control samples. Heatmap of the correlation between proteins and Medulloblastoma (Medullo), control (CTR), total CSF (Total), microvesicle (Mv), exosome (Ex) and proteins captured by peptide library beads (CPLLs) traits. The grade of Spearman's correlation coefficient ranged from – 0.8 (blue) to 0.8 (red). Tree dendograms at top and left of the heatmap show the un-supervised cluster analysis. The co-expression modules identified are reported on the left of the heatmap. Besides, the statistically significant proteins indentified in each comparison are highlighted in red on the right of the heatmap.

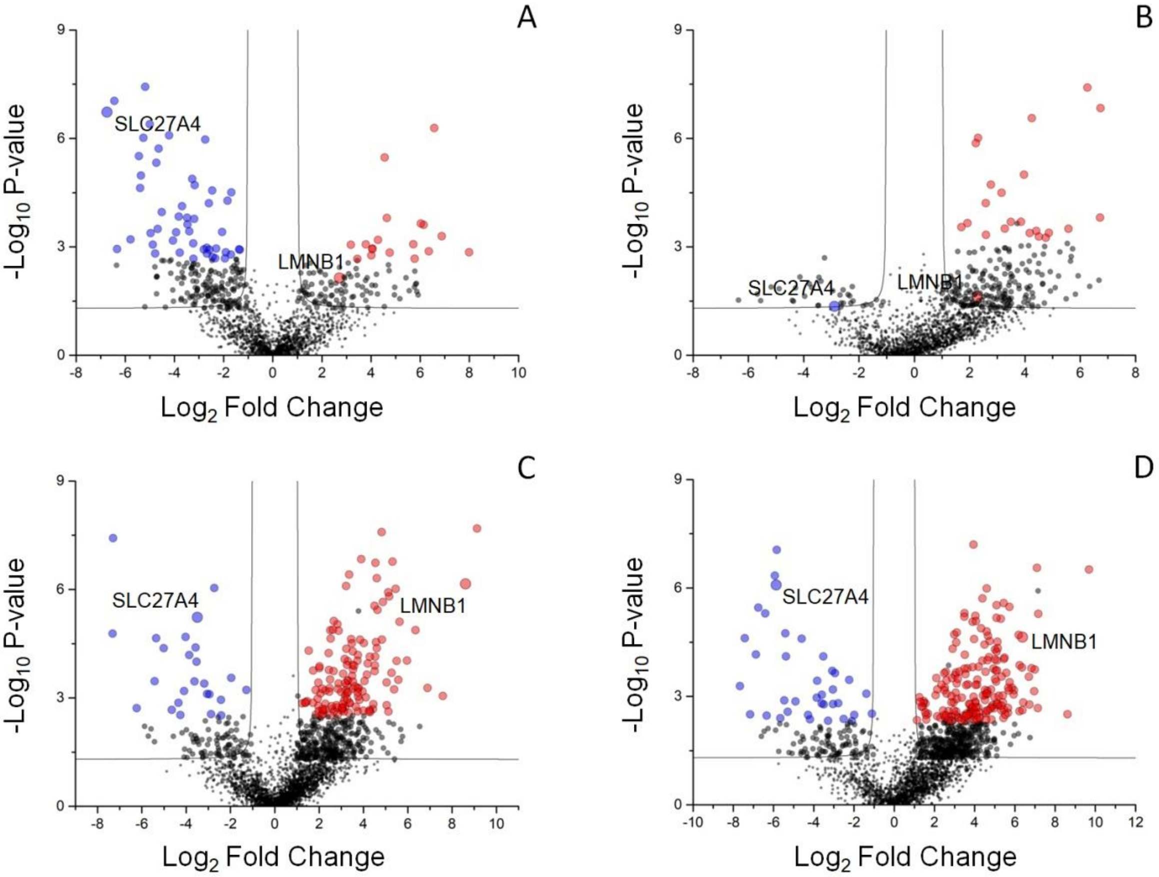

Figure S6. Volcano plot of univariate statistical analysis of CSF from EVD of MB and control samples. The plots are based on the fold change (log2) and their P-value (–log10) of proteins identified in the comparison of Medulloblastoma and control samples for total CSF (A), proteins captured by peptide library beads (CPLLs) (B), microvesicle (C), and exosome (D) fractions. Red, blue, and black circles indicate respectively the proteins with statistically significant up-regulation in Medulloblastoma or control samples, and the non statistically significant. Black line indicate the statistically significant cut-off.

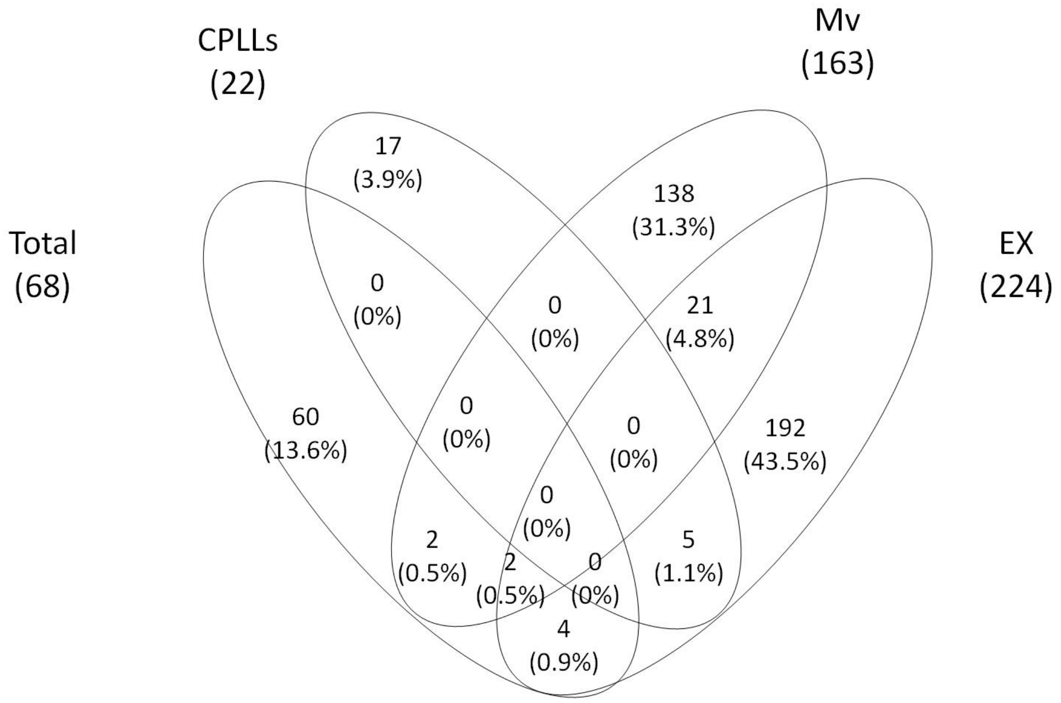

Figure S7. Venn diagram of statistically significant proteins identified in CSF from EVD of MB and control for each comparison. Venn diagram shows common and exclusive statistically significant proteins of total CSF (Total), microvesicle (Mv), exosome (Ex) and proteins captured by peptide library beads (CPLLs) fractions identified by univariate statistical analysis of Medulloblastoma and control samples. The numbers represent the distinct proteins in the overlapping and non-overlapping areas.

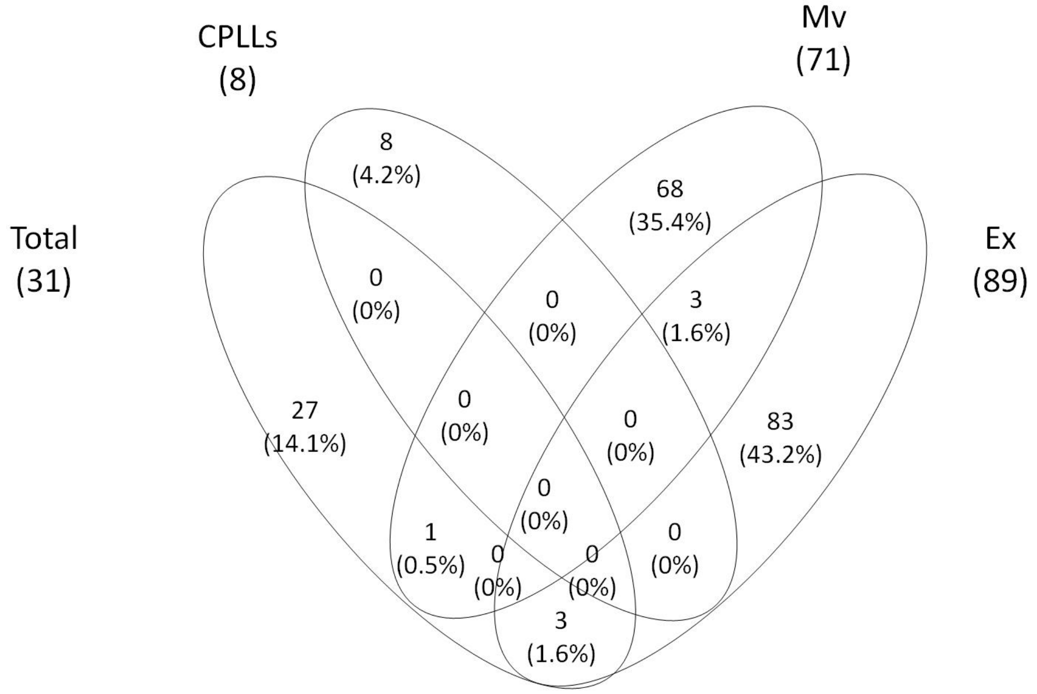

Figure S8. Venn diagram of highlighted proteins in CSF from EVD of MB and control by machine learning. Venn diagram shows common and exclusive proteins highlighted by Support Vector Machine (SVM) learning analysis in total CSF (Total), microvesicle (Mv), exosome (Ex) and proteins captured by peptide library beads (CPLLs) fractions of Medulloblastoma and control samples. The numbers represent the distinct proteins in the overlapping and non-overlapping areas.

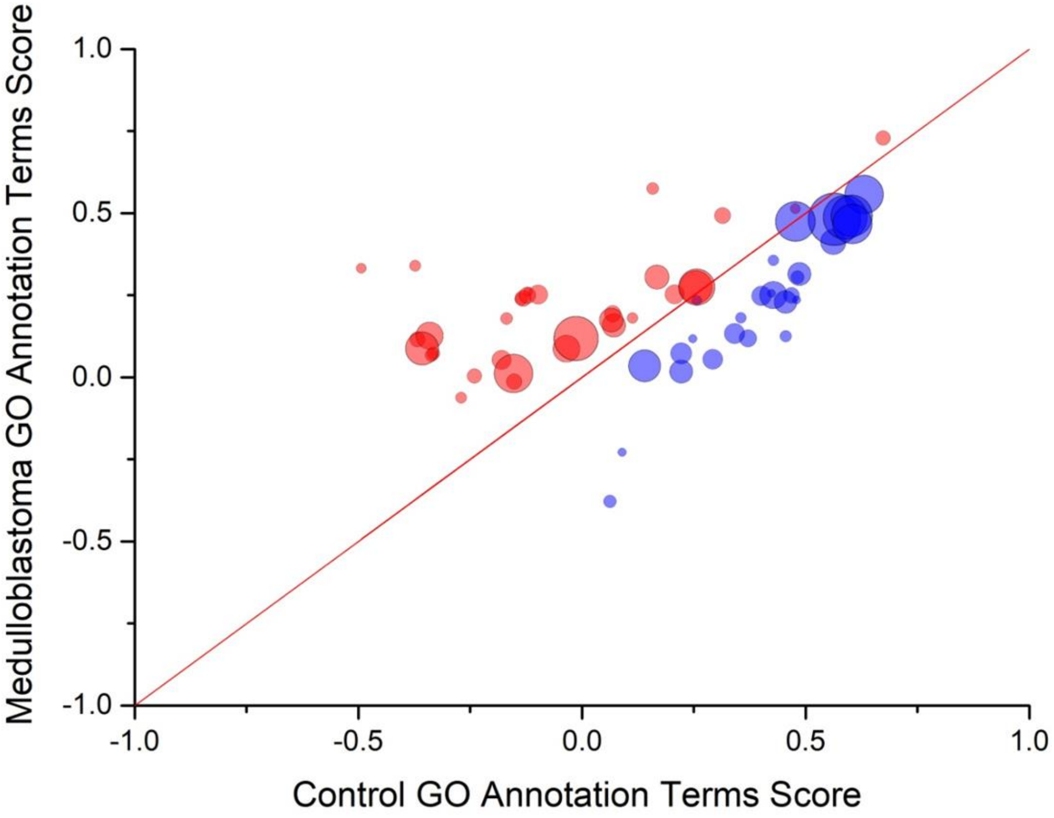

Figure S9. Two dimension Gene Ontology annotation enrichment analysis. Plot shows the enriched signatures in MB and control samples. In the graph, the points located on the straight line passing through the coordinates (1x,1y) and (-1x,-1y) are the equally enriched signatures, while those above or under this line are positively enriched in Medulloblastoma (red circles) or control (blue circles), respectively (see detail in Supplemental Table 3).
